# Stress Dependent Biofilm Formation and Bioactive Melanin Pigment Production by a Thermophilic *Bacillus* Species from Chilean Hot Spring

**DOI:** 10.3390/polym14040680

**Published:** 2022-02-10

**Authors:** Cathalina Marín-Sanhueza, Alex Echeverría-Vega, Aleydis Gómez, Gustavo Cabrera-Barjas, Romina Romero, Aparna Banerjee

**Affiliations:** 1 Escuela de Ingeniería en Biotecnología, Facultad de Ciencias Agrarias y Forestales, Universidad Católica del Maule, Talca 3466706, Chile; cathalina.marin@alu.ucm.cl; 2 Centro de Investigación de Estudios Avanzados del Maule, Vicerrectoría de Investigación y Posgrado, Universidad Católica del Maule, Talca 3466706, Chile; aecheverria@ucm.cl; 3 Centro de Biotecnología de los Recursos Naturales (CENBio), Facultad de Ciencias Agrarias y Forestales, Universidad Católica del Maule, Talca 3466706, Chile; agomez@ucm.cl; 4 Unidad de Desarrollo Tecnológico (UDT), Universidad de Concepción, Av. Cordillera 2634, Parque Industrial Coronel, Coronel 3349001, Chile; g.cabrera@udt.cl; 5 Centro Nacional de Excelencia Para la Industria de la Madera (CENAMAD), Pontificia Universidad Católica de Chile, Vicuña mackena 4860, Santiago 7820436, Chile; 6 Laboratorio de Investigaciones Medioambientales de Zonas Áridas (LIMZA), Depto. Ingeniería Mecánica, Facultad de Ingeniería, Universidad de Tarapacá, Arica 1020000, Chile; rominaromero@udec.cl

**Keywords:** extremophile, *Bacillus*, biofilm, pellicle, pigment, melanin, antioxidant

## Abstract

Thermophilic bacteria able to survive extreme temperature stress are of great biotechnological interest due to their extracellular production of bioactive molecules as a part of a survival strategy, or by intracellular modifications. In the present study, thermophilic *Bacillus haynesii* CamB6, isolated from a Chilean hot spring, was studied for the formation of different stress response molecules. The polymeric pigment produced by the bacterial strain was characterized by different physicochemical techniques. On exposure to ranges of temperature (50–60 °C), pH (5.0–7.0), and sources of nitrogen and carbon (1–5 g·L^−1^), the bacteria responded with a biofilm network formation in a hydrophobic polystyrene surface. Biofilm formation under fed-batch conditions was also statistically validated. The bacteria showed a planktonic pellicle network formation in the presence of induced hypoxia and salinity stress (19.45 g·L^−1^) under static conditions. Salinity stress also resulted in the intracellular response of brown pigment production. The pigment was structurally and functionally characterized by UV-Vis absorbance and the presence of different characteristic peaks via FTIR analysis (bacterial pyomelanin fingerprints) were assessed. A high thermal stability and TGA profile indicated the brown pigment was a probable pyomelanin candidate. Micropyrolysis (Py-GC/MS) showed that isoprene, pyrrole, benzene, pyridine, and their derivatives were the major components detected. In addition, acetic acid, indole, phenol, and its derivatives were observed. The absence of sulfocompounds in the pyrolyzed products agreed with those reported in the literature for pyomelanin. The pigment surface morphology was analyzed via SEM, and the elemental composition via EDS also demonstrated the similarity of the brown pigment to that of the melanin family. The pyomelanin pigment was observed to be bioactive with promising antioxidant capacity (H_2_O_2_, Fe^2+^) compared to the standard antioxidant molecules. In conclusion, *B. haynesii* CamB6 demonstrated the formation of several biomolecules as a stress response mechanism that is bioactive, showing its probable biotechnological applications in future.

## 1. Introduction

More than 80% of the surface of our planet is considered to be hostile to life and known as ‘extreme environment’ from an anthropogenic perspective. In many cases, what is considered as ‘extreme’ now was ‘normal’ nearly 2 billion years ago when the earth was hot and covered with anoxic hydrothermal vents [1]. Thus, thermophiles are considered to be the earliest form of life on our planet [2]. Microorganisms were naturally acclimatized to grow in such environmental conditions that are non-mesophilic, oligotrophic, and are subjected to different stress. They have adopted strategies via molecular evolution to protect themselves against extreme environments through the production of several unique and diverse bioactive molecules [3]. These microbial bioactive molecules are potentially valuable for biotechnological applications in the pharmaceutical, cosmetics, biomedicine, or food industries [4]. According to a previous report, nearly half of all Food and Drug Administration (FDA)-approved drugs are derived from natural products or derivatives, which are most likely produced by microorganisms [3]. Furthermore, thermophilic microorganisms are preferred for industrial productions of bioactive molecules for their adaptability to high temperature processes, thermostability, and ease in anaerobic fermentative processes [5].

Survival of thermophiles is based on a multi-dimensional integration of genomics, transcriptomics, and proteomics that include genomic stability, improved DNA repair system, smaller genome size, heat response proteins, thermozymes, and complex cell membrane structure [6,7]. Among all these adaptations to compensate for the deleterious impact of stress factors, quorum sensing (QS) plays an important role in the regulation of physiological processes in bacteria in any extreme environment [8]. QS helps function as a signaling pathway to produce an extracellular biofilm network of the bacteria that acts like a protecting sheath under extreme conditions [9]. The bacterial biofilm network is largely composed of exopolysaccharides (EPS), a carbohydrate polymer regularly used in pharmaceutical, food, and other industrial applications [10]. Thermophilic biofilms are also reported to have industrial applications in the activated sludge process of waste treatment [11], the electrochemical process of bioleaching [12], or biohydrogen production [13], and in the dairy industry. However, little is known about energy sources, environmental changes, and biofilm maturity, as the process is temperature dependent. Among the intracellular defense mechanisms of extremophiles, or specifically thermophiles, pigment production is an area that is relatively less studied. Pigments are reported to contribute strong antioxidant capacity against oxidative UV ray-mediated damage [14]. In the case of thermophiles, though pigment production, additional UV repair genes are reported [15,16,17], in-depth understanding of pigments and their impact on the defense or complete structural elucidation the molecule is lacking. This creates a promising avenue for the new application of thermophiles, as the thermophilic bacterial pigment molecules have theoretically demonstrated improved thermostability. As an alternative to synthetic pigments, bacterial pigments offer biodegradability and eco-friendliness offering opportunities for various biotechnological applications in food, pharmaceuticals, cosmetics, and textiles [18]. Melanins, one of the industrially important groups of bacterial pigments, are constituted of complex polyphenolic heteropolymers that include pyomelanin, eumelanin, and pheomelanin [19]. A number of pathways are known to covert tyrosine amino acid into melanin. Particularly, pyomelanins are produced by the accumulation of homogentisic acid (HGA), which is produced in the extracellular environment, auto-oxidized, and finally polymerized to form pyomelanin [19]. Still, extraction of bacterial pigments in relatively pure and concentrated forms remains a challenge.

Thermophiles, optimally growing at ~55 °C, are generally found in the extreme temperature environments of hot springs fumaroles, hydrothermal vents, geysers, or deserts [20]. As well as high temperatures, these extreme habitats also often have extreme pH or high salt concentrations [20]. As discussed earlier, the study of microorganisms inhabiting such ecosystems is interesting from a biotechnological point of view, as they produce several bioactive and thermostable molecules. In Chile particularly, extremophiles are reported from several hostile ecosystems in Altiplano, the Atacama Desert, Andean mountains, Patagonia, and Antarctica [21]. In this study, a thermophilic bacterium *Bacillus haynesii* CamB6, isolated from a Chilean hot spring, was studied for the formation and production of different stress response molecules including biofilm, pellicle cells, and pigments. Nutrient optimization for biofilm production was performed and biofilm formation was statistically validated. We elucidate the cellular morphology of stress-dependent pellicle cells. Finally, a stress response of bioactive, pigment molecules is structurally explained, along with its functions.

## 2. Materials and Methods

### 2.1. Sampling and Microorganism

The sampling site for this present work was the Campanario hot spring, located at the Andean Mountains of the Maule region, Chile (35°56′23″ S 70°36′22″ W). The sample water at the study site was slightly acidic (pH 5.82) with a surface water temperature of 56.4 °C. The water sample was collected using standard protocol. Serial dilution of the water sample was performed in Nutrient agar (NA) (Difco) media (pH 5.80) and incubated for growth at 55 °C for 72 h. The white, mucoid colony (characteristic of biofilm formation) of isolate CamB6 was further chosen for this study. 16S sequencing (using universal primers) indicated the isolate to show 100% similarity with *B. haynesii* and was further deposited in Genbank with accession number MZ298610. One factor at time (OFAT) optimization was performed to determine the best nutrient source for the growth of the isolate. The chosen nutrient was 1% C/N supplementation with carbon sources xylose, mannose, glucose, sucrose (all are standard sugars; SigmaAldrich), and nitrogen sources yeast extract, casein hydrolysate, and ammonium sulphate (Difco).

### 2.2. Biofilm Formation, Staining, and Quantification

Observing the mucoid colony appearance, a characteristic feature of biofilm production, a biofilm formation study of *B. haynesii* CamB6 was performed as earlier reported by Banerjee et al. [22] with some small modifications. For this, cells were inoculated in 50 mL nutrient broth (NB) (Difco) for 2 days at 55 °C with continuous shaking of 150 rpm (LM-450D BIOBASE, Jinan, China). Fifty microliters of the logarithmic phase culture (10^8^ CFL mL^−1^) was transferred to 96-well microplates and 250 µL of fresh culture broth was added. The microplate was further incubated for 24 h at 55 °C (BJP-H50 BIOBASE, Jinan, China). The design of biofilm formation media consisted of a factorial design formulated in Minitab [23], which include changes in nitrogen and carbon source concentration (1–5%), pH (5.0–7.0), and temperature differences (50–60 °C). The chosen nitrogen source and carbon source for this study were yeast extract and glucose respectively, based on the best observable growth. The biofilm was maintained for 4 days (appearance of visible biofilm formation on clear microplates) by discarding the planktonic cells from the microplate every 24 h and adding 250 µL of fresh broth.

Biofilm staining and quantification were performed according to the standard protocol of bacterial biofilm formation in the 96-well microtiter plate by Coffey and Anderson [24] with some small modifications. Briefly, the culture broth was discarded after 4 days by inverting the microplate with gentle shaking. The microplate was rinsed twice with Milli-Q water in a tray with moderate shaking, followed by tissue paper absorption. One hundred and twenty-five microliters of 0.5% crystal violet solution (Sigma, Darmstadt, Germany) was added to each well and allowed to stand for 10 min, which was then discarded by inverting the microplate and rinsing it with Milli-Q water in a tray twice. It was allowed to dry completely. Quantitation was accomplished by adding 150 µL of 30% acetic acid (Merck, Darmstadt, Germany) to each well and allowing it to stand for 10 min. This sample was transferred to an optically clear, flat-bottomed, sterile microplate. The optical density of the samples was measured at 550 nm using a Mobi-Microplate Spectrophotometer (μ2 MicroDigital, Seoul, Korea).

### 2.3. Pellicle Competition Analysis

The formation of pellicles was carried out in culture tubes under static conditions [25,26] for NB media (Difco), Luria Bertani (LB) (Difco), and Marine Broth (MB) (Difco), separately. For this, *B. haynesii* CamB6 cells in the logarithmic phase (10^8^ CFL mL^−1^) were inoculated in 15 mL of cultures in the culture test tubes (1:5, *v/v*), which were incubated at 55 °C for 4 days (BJP-H50 BIOBASE, Jinan, China). The cultures were visually examined for film formation at the air–liquid interface. For the observation of the pellicle cells and their morphology, scanning electron microscopy (SEM) was applied. For this, the pellicles were thoroughly washed with 0.1 M phosphate buffer (pH 7.4). The samples were fixed in 2.5% glutaraldehyde solution (Sigma) and were further dehydrated on an increasing concentration of 30, 40, 50, 60, 70, 80, 90, and 100% ethanol. Critical point drying was determined with CO_2_ (QUORUM K850, East Sussex, UK). The samples were placed in a 10 mm stub, covered with double beam carbon tape, and were made conductive with gold coating (SPI Supplies, West Chester, PA, USA) to observe under SEM (JEOL JSM 6380LV, Tokyo, Japan).

A 50 μL bacterial culture for each treatment was stained with LIVE/DEAD™ BacLight™ kit (ThermoFisher Scientific, MA, USA). SYTO 9 green only stains live cells in a fresh sample, whereas propidium iodide red only stains dying cells, i.e., cells under stress. SYTO 9 was used to verify live bacteria in the biofilm [27]. The stained cells were observed with a Leica Stellaris 5 Confocal microscope (Leica Microsystems, Wetzlar, Germany) with excitation/emission for SYTO 9: 485/498, and Propidium iodide: 535/617.

### 2.4. Pigment Production Study

#### 2.4.1. Physicochemical Characterization of the Pigment

- UV-Visible Analysis

For the physicochemical characterization of the pigment, the solubility nature of the pigment was tested in different pure solvents. From the obtained results, the aqueous pigment solution was examined with UV-Visible (UV-Vis) spectrophotometry, where the absorbance of the solution was recorded in a range of 180–600 nm in Mobi-Microplate Spectrophotometer (μ2 MicroDigital, Seoul, Korea).

- Fluorescence Property Analysis

A 20 μL aqueous pigment sample was taken on a glass slide and observed under a Leica Stellaris 5 Confocal microscope (Leica Microsystems, Wetzlar, Germany) with 405 nm excitation laser to determine the fluorescence property of the pigment.

- FTIR-ATR Analysis

The functional groups present in the pigment were analyzed using Fourier transform infrared spectroscopy (FTIR) with attenuated total reflection (ATR). The infrared spectrum of dry pigment powder was acquired in transmittance mode with an FTIR spectrometer (Jasco-4000, Jasco Analytical, Madrid, Spain). Spectra were recorded by pressing the samples into KBr granules in a 1:90 ratio, which were then scanned in the range of 4000–500 cm^−1^ with a resolution of 4 cm^−1^.

- Thermogravimetric Analysis

Thermal stability of the pigment was determined using a thermogravimetric analyzer (TGA) Cahn-Ventron 2000 (Cahn Scientific, Irvine, CA, USA) with a microprocessor-driven temperature control unit and thermal analysis data station. An approximately 5 mg powder pigment sample was placed in an aluminum sample pan with a temperature range from 25–600 °C at a heating rate of 10 °C min^−1^ under an N_2_ gas flow of 50 mL min^−1^.

- SEM-EDS Analysis

The microstructure of the pigment molecule was examined employing SEM. For this, the dry pigment sample was placed in a 10 mm stub, and covered with double-beam carbon tape and gold-coated (SPI Supplies, West Chester, PA, USA) to make it conductive. The stub was further observed with SEM (JEOL JSM 6380LV, Tokyo, Japan). At similar time, as mentioned in Section 3.3, the fresh pigment, being produced by the cells, was also observed. Elemental composition (C, O, P, S, N) of the pigment was analyzed using SEM coupled with Energy Dispersive Spectroscopy (EDS) (Oxford Instruments, High Wycombe, UK).

- Pyrolysis Gas Chromatography Analysis (py-GC/MS)

Finally, micropyrolysis coupled to mass spectrometry analysis (Py–GC/MS) was performed to examine the pyrolysis products during thermal degradation of the developed material. This analytical technique employs heat to break the complex and higher molecular weight compounds into smaller fragments. The Py-GC/MS experiments were carried out at 770 °C for 6 s in a micropyrolysis system (EGA/PY-3030D, Frontier Laboratories) interfaced with a gas chromatograph (GC-2010 Plus, Shimadzu) equipped with a single quadrupole mass spectrometry detector (QP 2010 Ultra, Shimadzu). The interface line was kept at 300 °C in all the experiments, and the pyrolysis products (1:50 split ratio) were separated in a Zebron-5MS capillary column (60 m × 0.25 mm × 0.25 µm; Phenomenex, Torrance, CA, USA). The injector and detector temperatures were kept at 250 °C and 280 °C, respectively. The initial GC oven temperature was 40 °C, which was held for 1 min before heating to 300 °C at 5 °C min^−1^, and it was finally maintained for 10 min. Once separated, the pyrolysis products were analyzed in an MS detector (70 eV ionization) within a m/z range of 20–500. The identification of compounds in the Py-GC/MS spectra was carried out by considering a minimum cut-off score of 80% in the National Institute of Standards and Technology (NIST) mass spectral database.

#### 2.4.2. Antioxidant Activity Determination

Antioxidant activity of the pigment was determined in different concentrations of aqueous pigment solution (0.1, 0.5, 1.0, 2.5, and 5.0 mg·mL^−1^). For hydrogen peroxide scavenging activity analysis [28], for each of the concentrations, 50 μL of sample, 120 μL of 0.1 M phosphate buffer (pH 7.4), and 30 μL of H_2_O_2_ were added (A1). The negative control for the study was H_2_O instead of sample (A0), while H_2_O instead of H_2_O_2_ was used to obtain results of the sample without any activity (A2). As a positive control, standard ascorbic acid (Sigma) was used. The assay set was dark incubated at ambient temperature for 15 min and the absorbance at 230 nm was measured in Mobi-Microplate Spectrophotometer (μ2 MicroDigital, Seoul, Korea). To determine the ferrous metal ions chelating activity [29] for each of the aqueous pigment concentrations, with 100 μL of sample, 10 μL of 2 mM FeCl_2_, and 40 μL of 5 mM ferrozine were added (A1). The negative control was H_2_O instead of sample (A0), as mentioned earlier, while H_2_O instead of 2 mM FeCl_2_ was used to obtain no activity (A2). In this case, as a positive control, EDTA (Merck) was used. After a dark incubation of 10 min at ambient temperature, the absorbance of the assay set was recorded at 562 in Mobi-Microplate Spectrophotometer (μ2 MicroDigital, Seoul, Korea). Both the % hydroxyl radicals (^•^OH) scavenging and % Fe^2+^ chelation were determined according to the following equation:

(1) $$\left[1-\frac{\left(A1-A2\right)}{A0}\right]\times 100$$

### 2.5. Statistical Analsysis

In this study, the tools used for statistical analysis are Minitab (State College, PA, USA) [23], MS-EXCEL version 2016, and Origin software version 8.5 (Northampton, MA, USA). All the experiments were performed in triplicates to obtain the mean values. Student’s *t*-tests were performed and differences were considered significant *p* < 0.05.

## 3. Results and Discussion

### 3.1. Biofilm Formation Study

**Figure 1:**
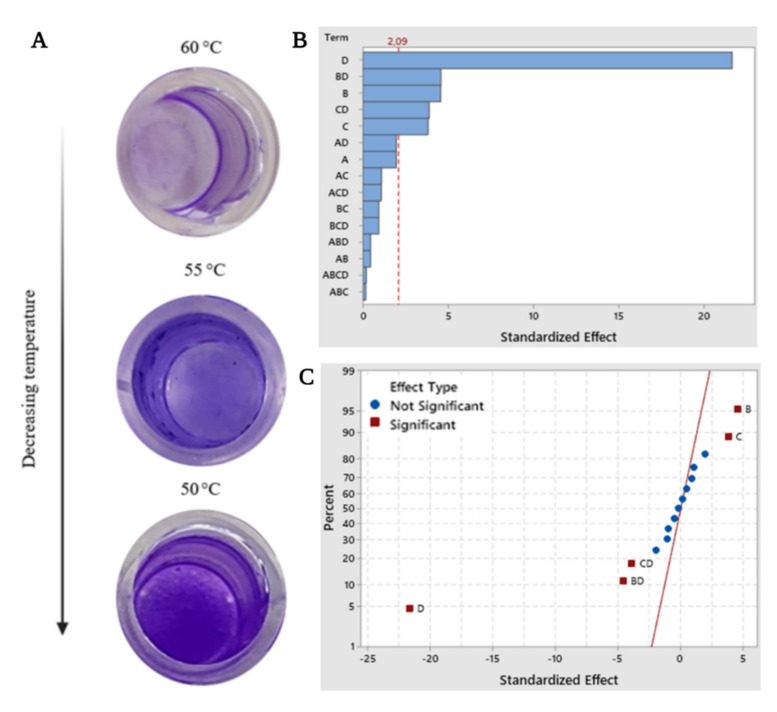
(**A**) Biofilm formation in 96-well microtiter plates after 96 h of incubation showing temperature dependence, factorial analysis of biofilm production (A = glucose as carbon source, B = yeast extract as nitrogen source, C = pH, and D = Temperature, with response in biofilm production value α = 0.05); where (**B**) Pareto chart of the standardized effects, and (**C**) normal plot of the standardized effects.

Biofilms formed by the bacteria as a part of their survival strategy have a significant impact in environmental, industrial, and medical applications; however, their formation can be affected by a series of culture parameters [30]. In our study, according to the result of the factorial analysis of biofilm formation by *B. haynesii* CamB6, the factors with a significant effect on the production of biofilms are found to be temperature (*p* < 0.000) (Figure 1A), pH (*p* < 0.001), and nitrogen (*p* < 0.000), and the interactions between the factors temperature–pH (*p* < 0.001), and temperature–nitrogen source (*p* < 0.000). Figure 1B shows the Pareto chart of standardized orders of all the effects. Along with the decreasing trend of temperatures for biofilm formation (60, 55, and 50 °C), at 50 °C, an absorbance maximum of 2.496 was recorded (cultured with 1 g·L^−1^ of yeast extract as a nitrogen source at pH 7.0) indicating the optimum biofilm formation by isolate CamB6. It can also be observed that the temperature alone demonstrated the most influential effect, with 50 °C being the optimum in the study carried out. It is also worth mentioning that the higher the temperature, the lower the formation of biofilms observed. While carbon source (specifically glucose) is considered the main factor for stable biofilm formation [31], it is interesting to highlight that the incorporation of a carbon source glucose in the culture medium has almost no influence on the formation of biofilms between the measured ranges (1–5% of carbon source, pH 5.0–7.0, temperature 50–60 °C), giving a *p*-value of 0.064 (>0.05). To the contrary, CamB6 is impacted by the nitrogen source for biofilm formation; while earlier *Xanthomonas oryzae* pv. *oryzae* showed inhibition to biofilm formation in the presence of a nitrogen source [32]. Furthermore, it can be detected from Figure 1B,C that the standardized value of the influence of carbon is below the minimum value of significance (dotted line). Additionally, in Figure 1C, showing the normal plot of the standardized effects, the effects that are significant (further from the red line) are presented in a red square, where α = 0.05. As mentioned above, the normal plot also confirms that the greatest influence on the formation of biofilms was temperature. Previously conducted studies indicate that pH plays a fundamental role in the formation of bacilli biofilms [33]. Similar to our results, pH 7.0 was also recently reported to be optimal for *B*. *vallismortis* biofilm formation [33]. Regarding the temperature factor, similar to our result, in a recent work by Kilic and Cihan [34], thermophilic *B*. *pumilus* also formed optimum biofilm at 50 °C with the highest attachment of sessile cells to multi-well polystyrene plates. In general, the temperature. range of favorable biofilm formation for thermophilic bacilli is 50–65 °C [35], which is also supported by our study. Overall, temperature, pH, and the composition of the medium vary between species: this may be due to each of them seeking the most efficient way to adhere to a substrate and colonize under an extreme environment. To interrelate this fact, in this study, the extreme temperature of 50 °C influenced thermophilic *B. haynesii* CamB6 to adhere better to the hydrophobic polystyrene surface of the solid–liquid interface and establish biofilm molecules under fed-batch conditions. To our knowledge, this study is also the first approach to understand biofilm formation by *B. haynesii* under varying culture conditions.

### 3.2. Pellicle Competition Analysis

**Figure 2:**
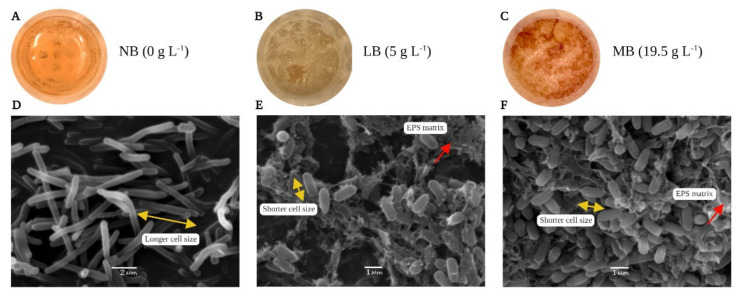
Pellicle formation by *B. haynesii* CamB6 by at air–liquid interface (**A**) NB medium, (**B**) LB medium, (**C**) MB medium; SEM photomicrographs of pellicles at 10,000×, (**D**) NB medium, (**E**) LB medium, (**F**) MB medium.

Pellicles are formed by the bacteria at the air–liquid interface under reduced oxygen availability or due to oxidative stress [36]. Pellicle formation by *B. haynesii* CamB6 was carried out in three different culture media with different salt compositions; NB (salinity = 0 g·L^−1^), LB (salinity = 5 g·L^−1^), and MB (salinity = 19.5 g·L^−1^). The results show that after 96 h, the MB medium produced a visibly thicker and uniform formation of pellicle cell network at the air–liquid interface (Figure 2C), compared to those grown in LB medium (Figure 2B). Pellicles formed in both MB and LB medium have shown characteristic slimy appearance with numerous thread-like structures of connecting clumps as mentioned earlier by Rajitha et al. [37] for marine *B. haynesii* isolate grown in Zobell marine broth. In this study, the firm pellicles formed in MB medium were also found to be pigmented a brownish color. In an earlier report, brownish pigment is reported in the pellicle formation by a deep-sea *Pseudoalteromonas* isolate and the pigment was pyomelanin-like compound [19]. The cells grown in NB medium demonstrated almost no formation of pellicle network (Figure 2A). This is may be because NB medium was devoid of any salt.

Pellicles, also known as air–liquid biofilm, are reported to be formed by the *Bacillus*, *Pseudomonas*, and *Vibrio* groups of bacteria [38]. Bacterial cell morphology in a pellicle is different from that of the planktonic cells. Planktonic cells exist independently, whereas pellicle cells generally show aggregation [39]. The three-dimensional architecture of pellicles is reported to be built on an extracellular polymeric matrix or EPS network [38]. In the present study, SEM analysis was performed to understand the cellular morphology, organization of the cells, pellicle matrix, and pellicle network. Figure 2E,F shows the difference in the density of the pellicles formed in LB and MB medium. As *B. haynesii* CamB6 grown in NB medium did not show any visible growth at the air–liquid interface; from Figure 2D, planktonic cell growth can be well observed with visibly longer cell size (size in yellow arrow), no polymeric network, and no cellular aggregation. MB medium with more salt content than LB medium revealed more cellular aggregation and less presence of polymeric matrix (polymeric network in the red arrow). In both cases, the polymeric networks were observed to be thread-like, hairy structures that connected different cells, and the cell sizes also visibly decreased during pellicle formation. Interestingly, cells grown in LB medium were seen to be more entrapped in an EPS matrix compared to MB medium that had a larger number of cells. Tight aggregation of cells and complex network of the pellicle can be supported by the earlier report on marine *B. haynesii* isolate [37]. In general, it demonstrated characteristic aerotaxis [40], a rapid response to oxidative stress through the establishment of extracellular pigmented pellicles in media containing salinity stress.

**Figure 3:**
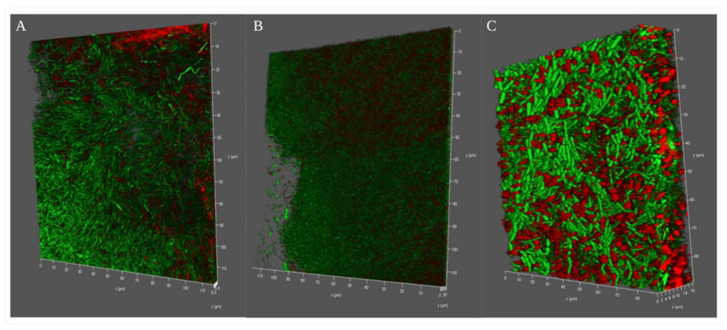
Confocal microscopy images of *B. haynesii* CamB6 competing against oxygen stress in different media and forming different depth of pellicles (Live–death images are shown in the green- or red-false-colored fluorescence channels merged); (**A**) NB medium, (**B**) LB medium, and (**C**) MB medium after 96 h of optimum pellicle formation.

To evaluate the broader relevance of our observations, we next examined the depth of pellicle formation, aerotaxis, polysaccharide network, and live cell–dead cell analysis through confocal microscopy. It was interesting to see the three dimensional depth of the pellicle formation in different media. While in the case of NB medium *B. haynesii* CamB6 cells formed ~5.5 μm of depth pellicle networks (Figure 3A), the maximum depth was observed for LB media with ~30 μm (Figure 3B). The MB medium with salinity stress demonstrated an 18 μm pellicle network (Figure 3C). Live bacterial cells embedded in a polysaccharide network were observed in all the three cases indicating polysaccharide production is a probable strategy during aerotaxis. This also confirm that *B. haynesii* CamB6 has maximum flagellar motility to air–medium interface for LB and MB media. Pellicle formation in *B*. *subtilis,* depending on motility, have been earlier reported by Hölscher et al. [41]. Biofilm or polysaccharide networks formed in LB medium also indicate most cells have an unaltered membrane integrity, being mostly green. The can be supported with the observation of Rodrigues et al. [42] for *Listeria monocytogenes* biofilm. In the case of the MB medium, more red cells indicate probable membrane damage under saline stress, which bacteria may fight with different strategies. To conclude, the pellicle competition study, *B. haynesii* CamB6 cells developed pellicle networks in the air–medium interface of the static culture depending on the media composition and oxygen availability.

### 3.3. Pigment Production Study

#### 3.3.1. Physicochemical Characterization of the Pigment

- UV-Visible Analysis

**Figure 4:**
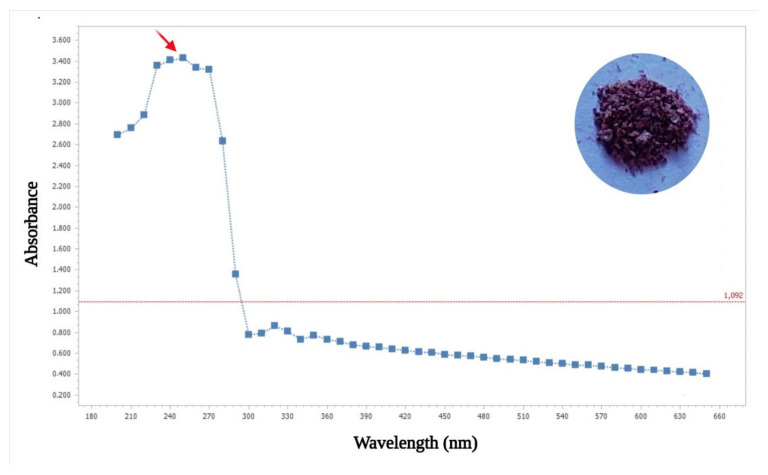
UV-Vis absorption spectrum (inset dry brown pigment compound) showing bacterial pyomelanin such as absorption with a peak at 250 nm (red arrow in the image).

The soluble dark brown pigment obtained from *B. haynesii* CamB6 grown in MB medium with an elevated salt level and heat stress was lyophilized before partial characterization (Figure 3A). For this, it was subjected to UV-Visible spectrophotometric analysis in the range of 180–600 nm, resulting in an absorbance peak at 250 nm (Figure 4). The peak was similar to that of melanin, specifically of the pyomelanin type, as a recent report indicated that melanin has a characteristic peak between 200–300 nm [43]. Furthermore, pyomelanin-type pigment molecule is also reported to have high values in the UV region, reaching a maximum between 250–280 nm wavelengths followed by a gradual decrease [44], similar to our result.

- Fluorescence Property Analysis

**Figure 5:**
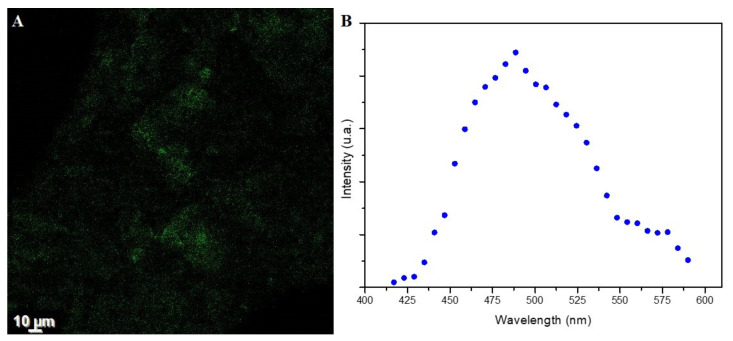
(**A**) Confocal microscopy images of *B. haynesii* CamB6 melanin and (**B**) fluorescence spectra of the sample.

The fluorescence of the brown pigment is demonstrated in Figure 5A and the spectra of *B. haynesii* CamB6 melanin have a Gaussian shape, showing a maximum absorption at 488.6 nm (Figure 5B). This behavior is similar to that previously reported from other melanin samples [45,46]. Some authors point out that such a profile can be due to the inherent structural heterogeneity, at the primary and secondary level, of melanin samples [46].

- FTIR-ATR Analysis

**Figure 6:**
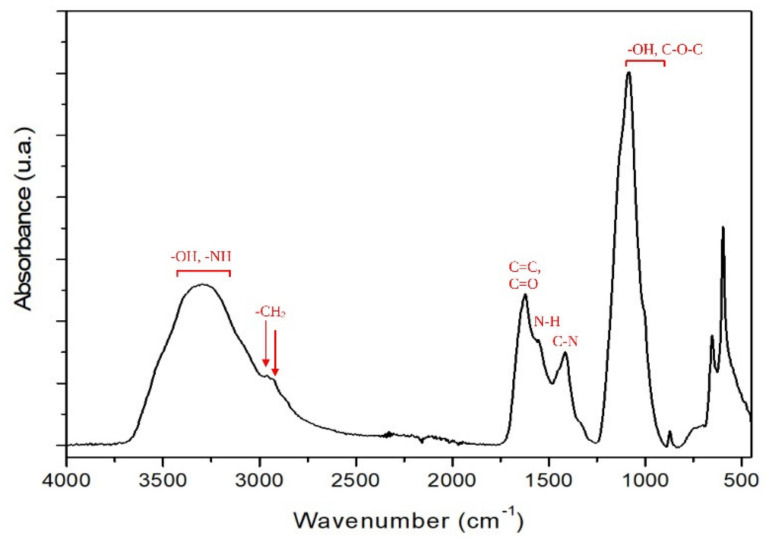
FTIR-ATR spectrum of the melanin pigment produced by *B. haynesii* CamB6.

FTIR is a valuable technique for identifying and characterizing different melanin structures. The infrared spectra of the melanin from *B. haynesii* is presented in Figure 6. In Figure 6, the broad bands in the 3700–3000 cm^−1^ region are caused by -OH and –NH stretching vibrations of the indole ring. The relative weak bands at 2954 cm^−1^ and 2927 cm^−1^ belong to –CH_2_ asymmetrical and symmetrical stretching. The sharp band at 1621 cm^−1^ is attributed to the aromatic C=C and C=O stretching vibrations [47]. The band at 1553 cm^−1^ is assigned to the N-H bending vibration, whereas the band at 1412 cm^−1^ is attributed to a C-N stretching vibration, which corresponds to the melanin indole structure [48]. Absorption bands between 1250 cm^−1^ and 900 cm^−1^ were observed in spectra of melanin isolated from different sources, which are ascribed to the stretching vibration of phenolic (–OH) groups [49]. Other absorption bands that appears between 1150–1100 cm^−1^ could be assigned the symmetric contraction vibration of C-O-C bond. Under 900 cm^−1^, the absorption bands are assigned to in-plane deformation of C-H bond, aromatic CH groups, or alkene CH substitution/conjugated systems [48]. In general, the FTIR spectra of *B. haynesii* CamB6 resemble other bacterial melanins [47].

- Thermogravimetric Analysis

**Figure 7:**
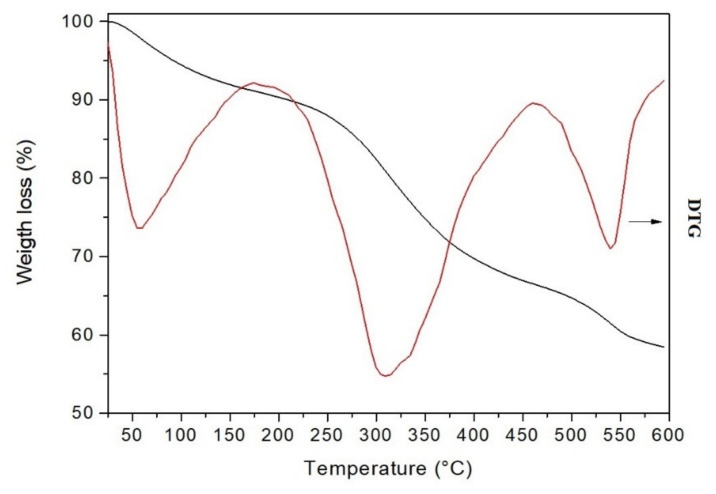
TGA (black line) and DTG (red line) curves of the melanin-like compound produced by *B. haynesii* CamB6 were recorded at 10 °C/min under N_2_ atmosphere (gas flow 50 mL/min).

Thermogravimetric analysis (TG) is a valuable technique for studying the thermal decomposition of compounds and determining their thermal stability. The results of TG-DTG (derivative thermogravimetry) analysis of melanin-like compound are summarized in Table 1 and presented in Figure 7. TG shows that *B. haynesii* thermogram is similar to that previously reported for *B*. *subtilis* melanin [50]. In the present work, the melanin compound undergoes three weight losses. The first thermal effect, ranging from 25–173 °C and peaking at 51 °C, shows an associated 8% weight loss. It corresponds to the release of melanin-bound intra- or intermolecular water [49]. The second effect occurs at a maximum decomposition rate of 309 °C, showing a weight loss of 24.8%. This weight loss is similar to that observed for other bacterial isolated melanin (21%) [50]. It probably corresponds to the decomposition of an aliphatic component in the melanin molecule, following the report by Ribera et al. [51] for fungal melanin compound. In addition, this effect occurs at similar temperatures reported for melanin isolated from black garlic, sepia ink, and *B*. *subtilis*, respectively [49,50]. However, it is lower than melanin isolated from other sources [47,51], indicating lower thermal stability. Simonovic et al. [52] previously suggested a relationship between melanin thermal stability, the polymer source, and their degree of polymerization. A third thermal effect can be observed from 463–600 °C, with T_Peak_ 541 °C, which may be due to the decomposition of aromatic compounds [51]. Furthermore, it is noticed that 58.4% of the total mass is retained at 600 °C. This amount is higher than other L-DOPA synthetic melanin reported that retained 40–50% at 500 °C [50].

- SEM-EDS Analysis

**Figure 8:**
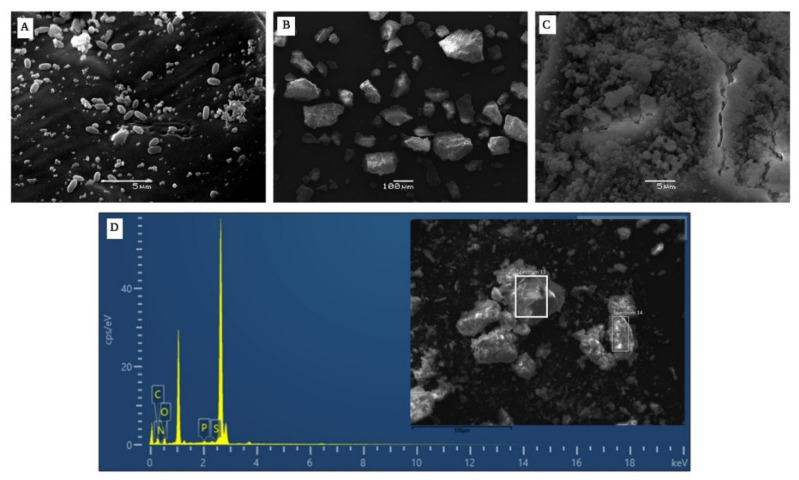
SEM images showing (**A**) *B. haynesii* CamB6 cells producing melanin-like compound in MB at 96 h of incubation, (**B**) extracted melanin-like compound 1000×, and (**C**) at 5000× showing high density, compact, amorphous deposit with no definite pattern. (**D**) Elemental analysis of the melanin showing the presence of C, O, N, P, and S (inset pigment at 1000×).

The morphology of the pigment produced by *B. haynesii* CamB6 in Figure 8B,C, resembles the previous reports of melanin produced by other *Bacillus* groups of bacteria, where it is suggested that melanin generally displays a high density, amorphous deposit without any distinguishable pattern [50]. The cells producing pigment were found to be smaller, as with other stress conditions, as mentioned in the other analyses (Figure 8A). The elemental analysis of the pigment molecule carried out through EDS coupled with SEM resulted in a higher percentage of oxygen and carbon, followed by nitrogen, phosphorus, and sulfur (Figure 8D). Previous studies on melanin pigments obtained from marine Actinomycetes indicated 6–11% N content, 41% carbon content of 41.04%, and 1.5% sulfur content [53]. On the other hand, elemental analysis of *Azotobacter*-produced melanin showed C and N content of 47.72% and 6.90%, respectively [54]. These results were found to be similar to the pigment obtained in this work (Table 2), presenting similarity to the composition with other bacterial melanins.

- Pyrolysis Gas Chromatography Analysis (py-GC/MS)

**Figure 9:**
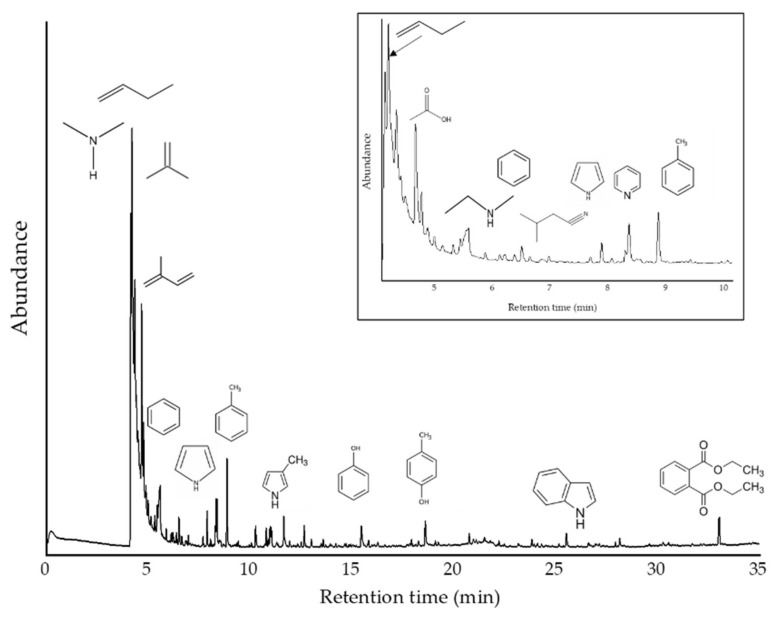
Py-GC/MS chromatogram for analyzed sample. Inset: Signal close-up to clarify thermal products in less than 10 min of retention times.

Py-GC/MS analyses were performed to determine the primary thermal decomposition compounds identified in the developed material (Figure 9). The fast pyrolysis performed (6 s) at 770 °C showed heterogeneity in the sample due to the different types of chemical compounds released after pyrolysis. In our sample, it was possible to identify small alkyl fragments or amine derivatives as the more abundant products at the beginning of the analysis (low retention times) due to their high volatility. Apart from high levels of low molecular weight gases (up to 4.33 min; %Area: 67.3), the most abundant pyrolysis product was isoprene (retention time: 4.77 min; 6.2%). Other prominent peaks corresponded to acetic acid (5.58 min; 4.13%), toluene (8.86 min; 3.27%), pyrrole (8.35 min; 2.78%), diethyl phthalate (33.02; 2.43%), *p*-cresol (18.59 min; 1.38%), phenol (15.45 min; 1.37%), butanenitrile, 3-methyl- (7.88 min; 1.18%), benzene (6.50 min; 1.12%), pyridine (8.28 min; 0.90%), and indole (25.52 min; 0.81%). These compounds are known markers of thermally degraded eumelanin pigment units [55,56,57,58]. All the remaining signals were reported with a percentage of abundance lower than 0.8%. The complete list of compounds detected is depicted in Table 3.

Therefore, the most abundant aromatic compounds were toluene, benzene, and ethylbenzene. Phenolic compounds were also detected as abundant compounds in the pyrolysate (phenol and *p*-cresol). It should also be noted that pyrrole molecules, pyridine and its derivatives, and, to a lesser extent, indole, were also found among the major products. The presence of acetic acid and phthalates also stood out. Other significant compounds were the -nitrile group (C≡N) derivatives. At the same time, no sulfur-derived compounds were detected in our Py-GC/MS analysis. However, the elemental analysis has shown about 2% of this element, so if compounds possess this element, they should be of much lower % area than those detected in this analysis (<0.15%), so they would be minority compounds. Alternatively, the pyrolysis products could be eluted during the first several minutes, where low molecular weight compounds, such as hydrogen sulfide, carbonyl sulfide, and methanethiol, have been previously found in the pyrogram of pheomelanin standard [59]. When the detected compounds were compared with those reported in the literature, they matched the chemical profiles found for natural melanins. Pyrograms in literature highlighted pyrrole, benzene, and their derivatives as main components. Acetic acid, indole, phenol, and its derivatives were also detected in the pyrolyzed products [60,61].

Subsequently, the compounds were grouped and analyzed according to their determining functional group: amines, hydrocarbons (alkyl), aromatic amines, acids, azo-compounds, nitrile compounds, aromatic, phenols, and esters. Accordingly, it was found that when normalizing the areas according to their total area percentage [62], the relative content of alkyl compounds is close to 60%, while the content of amines (linear, non-aromatic) is close to 14%. On the other hand, aromatic amines reach about 8%, aromatic compounds 5.2%, and acids 4.1%. Further down, with 2.8% phenolic compounds, 2.5% nitrile compounds, and 2.4% esters. Azo compounds (N=N) were found in smaller proportions (<0.3%). The observed thermal decomposition profile coincides with that reported for natural melanin in the literature, which the main compounds (depending on the precursor) overlap with those reported in this work. In a more detailed analysis of the data, it was observed that it is in agreement with the profiles reported for extracellular melanins (pyomelanin), where one of the significant characteristics was the presence of acetic acid, aromatic derivatives, and also the absence of sulfocompounds [47].

In general, the brown pigment molecule produced by *B. haynesii* CamB6 has displayed similarity with melanin, specifically pyomelanins. However, different pathogenic bacteria are reported to produce pyomelanin for a long time, which protect them against oxidative and UV damage including *Pseudomonas aeruginosa* of cystic fibrosis or urinary tract infection origin [63], *B*. *anthracis* [64]. Recently, bacteria from other ecosystems are also reported to produce this pigment, for example, a photosynthetic Rubrivivax [65]. In this study, the hot spring origin, thermophilic *Bacillus* species was shown to produce the pyomelanin-like molecule production against temperature and salinity stress (while grown in marine broth medium).

#### 3.3.2. Antioxidant Activity Determination

**Figure 10:**
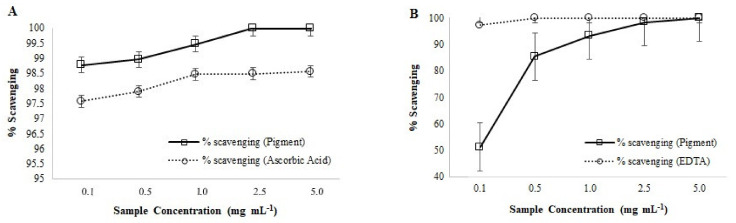
Dose-dependent antioxidant activities of the pyomelanin-like compound produced by *B. haynesii* CamB6; where (**A**) hydroxyl radicals (^•^OH) scavenging effects, and (**B**) ferrous (Fe^2+^) chelating capacity.

Melanin has been well recognized for its activity as reactive oxygen (ROS). A study of melanin obtained from *Aspergillus nidulans* showed oxidation of 5-thio-2-nitrobenzoic acid (TNB) by peroxide hydrogen (H_2_O_2_) and hypochlorous acid (HClO), indicating that synthetic melanin reacts with these compounds and reduces the amount of TNB to be oxidized [66]. Regarding the melanin (specifically pyomelanin-like) compound obtained in our study, it has demonstrated a maximum of 100% of the ^•^OH scavenging. Compared to this, commercial antioxidant ascorbic acid has displayed a maximum of 98.56% hydroxyl radical scavenging (Figure 10A). Both the tested ascorbic acid and pigment displayed % scavenging dose-dependently. However, the pigment solution was a more effective ^•^OH scavenger at all the tested concentrations. A similar kind of effectiveness of plant-origin melanin-like pigment (chestnut shell) has been earlier mentioned in Yao and Qi [67]. The hydroxyl radicals (^•^OH) are the most reactive ROS generated in the body, which react with almost all components of the cell, including nucleotides, proteins, and lipids [68]. Due to high toxicity, scavenging capacity towards these radicals is widely used as an important indicator to evaluate the antioxidant capacity of any compounds [67].

Regarding the antioxidant activity associated with the chelation of ferrous ions (Fe^2+^), it has been previously suggested that the melanin pigment has a peroxidation inhibitory capacity, which may be associated with its iron-binding property [67]. In that study it was mentioned that chelating agents can modulate the catalytic activity of the metal ions and serve as secondary antioxidants, therefore, EDTA (strong metal chelator) was used as a positive control [67], similar to our present work. In the present study, a maximum of 100% of the Fe^2+^ chelation was observed by the pyomelanin-like pigment and the pigment showed a linear increase in % Fe^2+^ scavenging with an increase in the pigment concentration. However, it was still significantly weaker in a dose-dependent way compared to the strong iron chelator EDTA (Figure 10B). This kind of dose-dependent linear increase in pigment activity, weaker compared to EDTA, was earlier reported by Li et al. [69]. The report was on animal-origin (sea urchin) polyhydroxylated 1,4-naphthoquinone pigments that are also brown. Interestingly, melanin also constitutes quinone derivatives [70].

Melanin pigments from different origins; fungi, animal retinal cells, plant shells, and bacteria are well known for their photoprotective activity as antioxidants [67,71,72,73,74,75]. In recent times, melanin nanoparticles are also being reported for increased shelf life as antioxidants [76]. The technological necessity of antioxidants in the food industry to have consumer-oriented functional foods is a well-known fact [77]. In addition, considering the negative impact of synthetic antioxidants in carcinogenicity, cytotoxicity, oxidative stress induction, and endocrine-disrupting effects [78], the search for natural antioxidants for industrial usage is important. In this study, thermophilic *B. haynesii* CamB6 intracellularly produced a pyomelanin-like compound against salinity and temperature stress that have demonstrated efficient scavenging activity of model hydroxyl radicals and ferrous ions.

## 4. Conclusions

In this study, the thermophilic *B. haynesii* CamB6 isolated from Campanario hot spring of Chile demonstrated the production of biofilm, pellicles, and brown pyomelanin-like pigment against a series of stress factors. The general factors that significantly influenced the biofilm formation on hydrophobic polystyrene surface were temperature, nitrogen source, and pH of the fed-batch growth condition. This organism demonstrated pellicle formation under static condition with oxygen availability (air–liquid interface) and salinity stress while cultivating in marine broth medium, rich in salts. The cells were found to be mostly viable in the pellicle network. The cellular morphology significantly changed under stress, being smaller in size and in network of EPS matrix formation. It also produced a pyomelanin-like pigment molecule under salinity and temperature stress demonstrating efficient hydroxyl radicals and ferrous ions scavenging activity. These properties indicate that the thermophilic strain can be further used as an industrial biocatalyst for antioxidant pigment production. However, further research is needed to purify the pigment molecule to find its exact nature in order to confirm the antioxidant property in vivo.

## Figures and Tables

**Table 1:** Thermogravimetric properties of melanin compound from *B. haynesii* CamB6.

Sample	Temperature (°C)			Weight Loss (%)
	T_Onset_	T_Peak_	T_End_	
Pyomelanin	25	51	173	8.8
	174	309	462	24.8
	463	541	600	8.1

**Table 2:** EDS elemental composition of the melanin-like deep brown pigment produced by *B. haynesii* CamB6.

Element	Weight%	Weight% Sigma	Atomic %
C	43.65 ± 5.05	0.83 ± 0.16	49.61 ± 3.13
O	42.66 ± 4.60	0.83 ± 0.20	37.60 ± 4.61
P	1.96 ± 1.19	0.09 ± 0.42	0.90 ± 0.56
S	2.16 ± 1.38	0.06 ± 0.03	0.95 ± 0.60
N	10.91 ± 2.14	1.33 ± 0.28	10.95 ± 1.98
Total	100		100

The result is indicating the mean value and the standard error of each experiment. The assay was performed in triplicates.

**Table 3:** Py-GC/MS detected compounds.

R.Time	Area (%)	Name	Classification	*m/z*
4.14	13.8	Dimethylamine	Amine	44, 45, 28, 42, 43
4.20	33.8	Propene	Hydrocarbon	41, 42, 39, 27, 40, 38
4.34	19.7	1-Propene, 2-methyl-	Hydrocarbon	41, 56, 39, 55, 28, 27
4.77	6.23	Isoprene	Hydrocarbon	67, 53, 68, 39, 40, 41
5.58	4.13	Acetic acid	Acid	43, 45, 60, 42
5.87	0.4	Isobutyronitrile	Nitrile	42, 68, 54, 41, 28
6.50	1.12	Benzene	Aromatic	78, 77, 51, 50, 52, 39
7.68	0.35	Ethenamine, N-methylene-	Amine	28, 55, 54, 27
7.88	1.18	Butanenitrile, 3-methyl-	Nitrile	43, 41, 27, 39, 68
8.28	0.74	Pyridine	Aromatic Amine	79, 52, 51, 50, 78
8.35	2.78	Pyrrole	Aromatic Amine	67, 39, 41, 40, 28
8.86	3.27	Toluene	Aromatic	91, 92, 65
10.26	0.91	Pyridine, 2-methyl-	Aromatic Amine	93, 66, 39, 92, 51
10.78	0.68	1H-Pyrrole, 3-methyl-	Aromatic Amine	80, 81, 53, 52, 51
10.89	0.27	Diazene, bis(1,1-dimethylethyl)-	Azo	57, 41, 29, 39
10.97	0.89	Isoamyl cyanide	Nitrile	55, 41, 43, 57, 54
11.03	0.82	1H-Pyrrole, 2-methyl-	Aromatic Amine	80, 81, 53, 52, 51
11.65	0.85	Ethylbenzene	Aromatic	91, 106, 51, 65, 77
12.65	0.84	Bicyclo [4.2.0]octa-1,3,5-triene	Hydrocarbon	104, 103, 78, 51, 50
13.00	0.39	Pyridine, 2-ethyl-	Aromatic Amine	106, 107, 79, 78, 52
13.47	0.15	1H-Pyrrole, 2,5-dimethyl-	Aromatic Amine	94, 95, 80, 93, 42
13.58	0.29	1H-Pyrrole, 3-ethyl-	Aromatic Amine	80, 95, 53, 94, 67
15.45	1.37	Phenol	Phenolic	94, 66, 65, 39, 40
18.59	1.38	*p*-Cresol	Phenolic	107, 108, 77, 79, 80
20.75	0.42	5H-1-Pyrindine	Aromatic Amine	117, 90, 89, 63, 39
25.52	0.81	Indole	Aromatic Amine	117, 90, 89, 63, 118
33.02	2.43	Diethyl Phthalate	Ester	149, 177, 150, 65

## Data Availability

Not applicable.

## Abbreviations

NA	nutrient agar
OFAT	one factor at time
NB	nutrient broth
LB	Luria Bertani
MB	marine broth
UV	ultra violet
UV-Vis	UV-Visible
FTIR-ATR	Fourier transform infrared spectroscopy-attenuated total reflection
TG	thermogravimetric analysis
SEM	scanning electron microscopy
EDS	energy dispersive spectroscopy
py-GC/MS	pyrolysis gas chromatography/mass spectrometry
EDTA	ethylenediamine tetraacetic acid
EPS	Exopolysaccharide
